# Early skeletal muscle mass decline is a prognostic factor in patients receiving gemcitabine plus nab-paclitaxel for unresectable pancreatic cancer: a retrospective observational study

**DOI:** 10.1007/s00520-023-07659-w

**Published:** 2023-03-02

**Authors:** Yukari Suzuki, Kei Saito, Yousuke Nakai, Hiroki Oyama, Sachiko Kanai, Tatsunori Suzuki, Tatsuya Sato, Ryunosuke Hakuta, Kazunaga Ishigaki, Tomotaka Saito, Tsuyoshi Hamada, Naminatsu Takahara, Ryosuke Tateishi, Mitsuhiro Fujishiro

**Affiliations:** 1 Department of Gastroenterology, Graduate School of Medicine, The University of Tokyo, Tokyo, Japan; 2 Division of Gastroenterology and Hepatology, Department of Internal Medicine, Nihon University School of Medicine, Tokyo, Japan; 3 Department of Endoscopy and Endoscopic Surgery, The University of Tokyo Hospital, 7-3-1 Hongo Bunkyo-Ku, Tokyo, 113-8655 Japan; 4 Department of Outpatient Chemotherapy, The University of Tokyo Hospital, Tokyo, Japan

**Keywords:** Gemcitabine plus nab-paclitaxel, Pancreatic cancer, Skeletal muscle mass, Sarcopenia

## Abstract

**Purpose:**

Patients with pancreatic cancer often have cancer cachexia at diagnosis. Recent studies suggested that loss of skeletal muscle mass was related to cancer cachexia, which hindered continuance of chemotherapy and could be one of prognostic factors in pancreatic cancer, however the association remains unclear in patients receiving gemcitabine and nab-paclitaxel (GnP).

**Methods:**

We retrospectively studied 138 patients with unresectable pancreatic cancer receiving first-line GnP at the University of Tokyo from January 2015 to September 2020. We calculated body composition in CT images before chemotherapy and at initial evaluation, and evaluated the association of both body composition before chemotherapy and its changes at initial evaluation.

**Results:**

Compared by skeletal muscle mass index (SMI) change rate between pre-chemotherapy and initial evaluation, there were statistically significantly differences in the median OS: 16.3 months (95%CI 12.3–22.7) and 10.3 months (95%CI 8.3–18.1) between SMI change rate ≥ -3.5% and < -3.5% groups (*P* = 0.01). By multivariate analysis for OS, CA19-9 (HR 3.34, 95%CI 2.00–5.57, *P* < 0.01), PLR (HR 1.68, 95%CI 1.01–2.78, *P* = 0.04), mGPS (HR 2.32, 95%CI 1.47–3.65, *P* < 0.01) and relative dose intensity (HR 2.21, 95%CI 1.42–3.46, *P* < 0.01) were significantly poor prognostic factors. SMI change rate (HR 1.47, 95%CI 0.95–2.28, *P* = 0.08) showed a trend to poor prognosis. Sarcopenia before chemotherapy was not significantly associated with PFS or OS.

**Conclusion:**

Early skeletal muscle mass decline was associated with poor OS. Further investigation is warranted whether the maintenance of skeletal muscle mass by nutritional support would improve prognosis.

## Introduction

The incidence of pancreatic cancer (PC) is increasing worldwide [1]. Despite surgery being the only curative treatment, 80–85% of patients present with an advanced stage [2, 3]. Immunotherapy has been investigated as one of treatment options, but systemic cytotoxic chemotherapy is still the standard of care for locally advanced or metastatic PC, including gemcitabine plus nab-paclitaxel (GnP) [4], and FOLFIRINOX (5-fluorouracil, leucovorin, irinotecan, and oxaliplatin) [5]. Despite the improvement of survival by those intense combination regimens, they are associated with adverse effects (AEs) and require appropriate patient selection.

Patients with PC, especially elderly patients, are often underweight and undernourished at diagnosis, with 50% reported to have cancer cachexia at diagnosis [6, 7]. Cancer cachexia is defined as a multifactorial syndrome defined by an ongoing loss of skeletal muscle mass that cannot be fully reversed by conventional nutritional support and leads to progressive functional impairment [8]. Recent studies suggested that loss of skeletal muscle mass was associated with cancer cachexia, which hindered continuance of chemotherapy, and can be one of prognostic factors of survival in PC [9–13]. However, it remains unclear whether sarcopenia at diagnosis or decline in skeletal muscle mass during chemotherapy is more prognostic of survival in PC, with various regimens such as FOLFIRINOX [14, 15] and GnP [16] being evaluated.

In this retrospective study, we investigated the association of both body composition before chemotherapy and its changes at initial evaluation of chemotherapy in patients receiving first-line GnP for unresectable PC.

## Methods

### Patients

Data on patients with unresectable PC who started GnP as first-line chemotherapy at the Department of Gastroenterology, the University of Tokyo from January 2015 to September 2020 were retrospectively studied. The analysis was based on follow-up information, which was received until April 2022. This study was approved by the ethics committee of the University of Tokyo Hospital.

All patients were histologically or cytologically diagnosed as pancreatic ductal adenocarcinoma and were diagnosed as locally advanced or metastatic diseases on CT. Chemotherapy was administered on days 1, 8 and 15 of a 28-day cycle, combined gemcitabine at 1000 mg/m^2^ and nab-paclitaxel at 125 mg/m^2^ [17].

### Data collection

We extracted data, including age, sex, height, weight, Eastern Cooperative Oncology Group performance status (ECOG PS), laboratory data (white blood cell with differential, albumin, C-reactive protein, carcinoembryonic antigen and carbohydrate antigen 19–9 [CA19-9]) from our prospectively maintained pancreatic cancer database and electric medical records in our hospital.

Body Mass Index (BMI) was calculated by dividing the weight (kg) by the square of the height (m), and the cutoff value was set at 22, the standard value in Japan. Neutrophil/lymphocyte ratio (NLR), Platelet/lymphocyte ratio (PLR) and modified Glasgow Prognostic Score (mGPS) were calculated from the above-mentioned data. The cutoff value of NLR and PLR was set by creating receiver operating characteristic (ROC) curve with a dichotomous variable divided by median overall survival (353.5 days) as the dependent variable.

In addition, we evaluated relative dose intensity (RDI) up to first 2 cycles, early tumor shrinkage (ETS) and presence of dose reduction at 1st cycle to analyze prognostic factors. RDI was calculated by dividing the actual dose by the standard dose of gemcitabine and nab-paclitaxel up to first 2 cycles, the cutoff value was set at the median. The standard dose was set at 125 mg/m^2^ for nab-paclitaxel and 1000 mg/m^2^ for gemcitabine, based on the results of the phase 3 study with metastatic pancreatic cancer [4, 17]. ETS was calculated from the maximum tumor diameter before chemotherapy and at initial evaluation according to RECIST 1.1, the cutoff value was set at 20% [18–20].

### Body composition assessment

**Fig.1:**
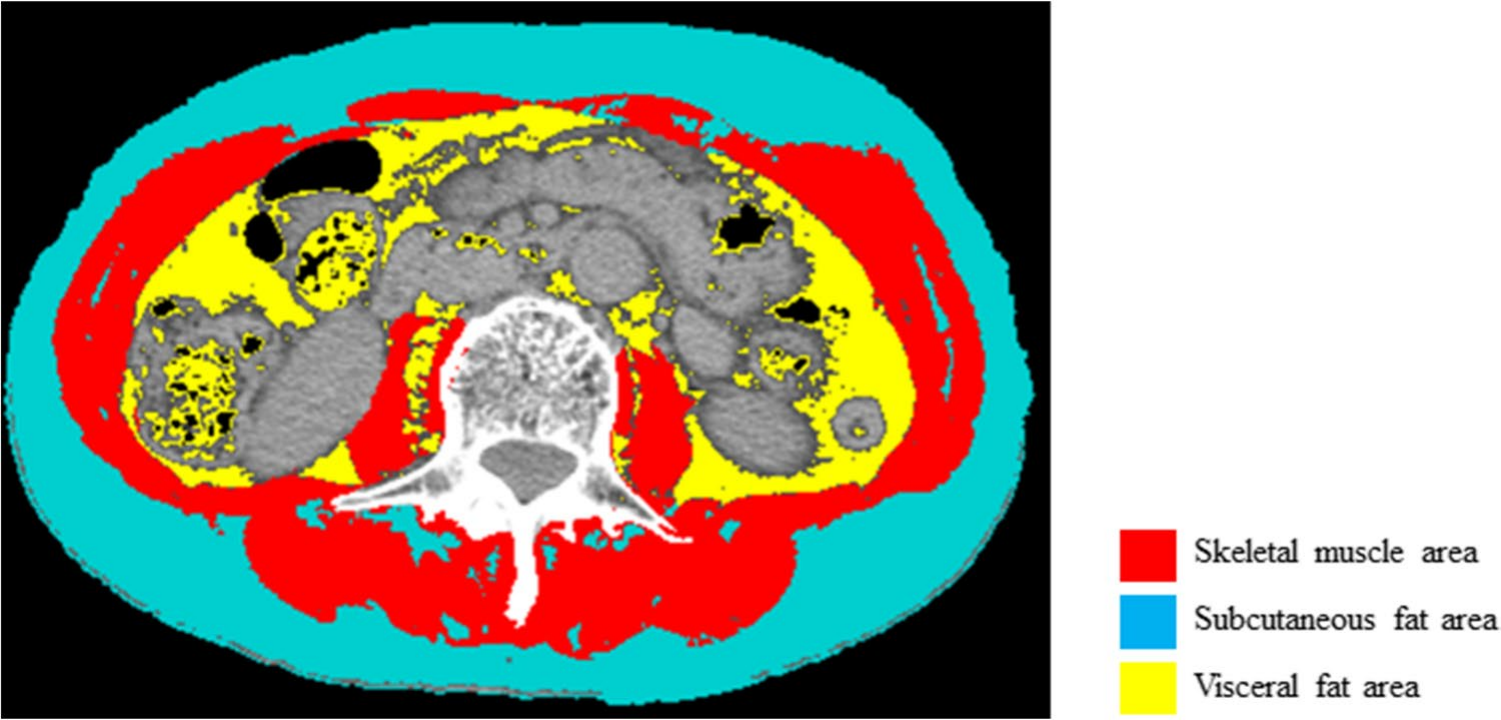
Assessment of body composition. The image illustrates the different proportions of skeletal muscle area (red), subcutaneous fat area (turquoise), and visceral fat area (yellow). Skeletal muscle area (cm^2^), subcutaneous fat area (cm^2^), and visceral fat area (cm^2^) at the level of the third lumbar vertebra in CT scan were quantified by using SliceOmatic medical imaging software. Skeletal muscle area highlighted red was quantified within -29 to 150 HU, subcutaneous fat area highlighted turquoise was quantified within -190 to -30 HU, and visceral fat area highlighted yellow was quantified within -150 to -50 HU

We calculated the skeletal muscle mass area (cm^2^), subcutaneous fat area (cm^2^) and visceral fat area (cm^2^) at the level of the third lumbar vertebra in CT images before chemotherapy introduction and at initial evaluation by using SliceOmatic medical imaging software (Tomovision, Canada) [21]. The ranges of tissue Hounsfield unit (HU) thresholds were within -29 to 150 HU for skeletal muscle mass area, -190 to -30 HU for subcutaneous fat area, and -150 to -50 HU for visceral fat area, as shown Fig. 1 [22]. Skeletal muscle area, subcutaneous fat area, and visceral fat area were normalized for height in meters squared (m^2^) and reported as skeletal muscle mass index (SMI) (cm^2^/m^2^), subcutaneous adipose tissue index (SATI) (cm^2^/m^2^), and visceral adipose tissue index (VATI) (cm^2^/m^2^). Visceral-to-subcutaneous fat area ratio (VSR) was calculated by dividing visceral fat area by subcutaneous fat area to assess for the presence of visceral obesity. SMI change rate (%) was calculated by subtracting SMI before chemotherapy from SMI at initial evaluation, and dividing by SMI before chemotherapy, and standardizing at 60 days.

Sarcopenia was defined as male SMI < 42 cm^2^/m^2^ and female SMI < 38 cm^2^/m^2^ based on the criteria proposed by the Hepatology Society of Japan [23, 24]. The cutoff values for VSR and SMI change rate were set by creating the ROC curve with a dichotomous variable divided by median overall survival as the dependent variable.

### Statistical analysis

We investigated the association of sarcopenia and changes in body composition during chemotherapy with progression free survival (PFS), overall survival (OS), AEs, and tumor response including response rate (RR) and disease control rate (DCR).

Both PFS and OS were calculated starting from the CT date of initial evaluation. PFS and OS were estimated using Kaplan–Meier method and survival curves were compared using log-rank test. Comparisons between two groups were evaluated using the Mann–Whitney U test for continuous variables and using the Fisher’s exact test for categorical data. AEs were evaluated according to CTCAE ver 4.0. Hazard ratios (HRs) with 95% confidence intervals (CIs) for OS and PFS were estimated by a Cox proportional hazards model to determine the independent prognostic factors. Factors with *p*-values < 0.20 in the univariable analyses were evaluated in the multivariable analyses. All tests were 2-sided, and *p*-value < 0.05 was considered statistically significant. Statistical analyses were performed using JMP version 16 software (SAS Institute Inc., Cary, NC).

## Results

### Patient characteristics

**Fig. 2:**
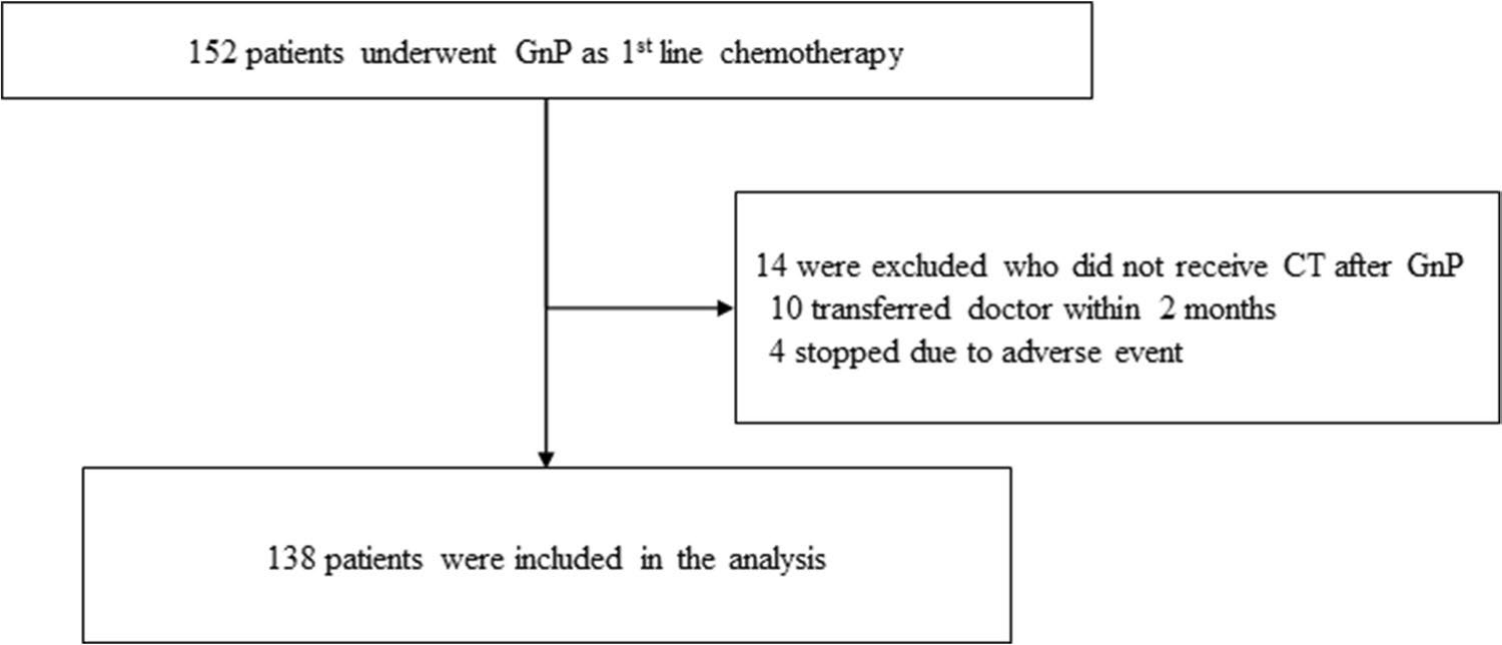
Patient flowchart. GnP; gemcitabine and nab-paclitaxel

Between January 2015 and September 2020, 152 patients started GnP as first-line chemotherapy, and pre-chemotherapy and initial evaluation CT scans were available in 138 patients. Fourteen patients who did not receive follow up CT evaluation were excluded from the analysis (Fig. 2). The median interval between pre-chemotherapy and initial evaluation CT scan was 61.5 days (interquartile range (IQR), 54–70). The median follow-up period was 13.1 months (IQR, 8.9–22.5). Baseline characteristics at chemotherapy introduction are summarized in Table 1. Median age was 67.5 years old (IQR, 59.7–74) and 80 patients (58.0%) were male. Distant metastasis was present in 97 patients (70.3%); liver in 62 (44.9%), lung in 20 (14.5%), lymph nodes in 38 (27.5%) and peritoneal dissemination in 21 (15.2%). ECOG PS was 0 in 76 patients (55.1%). The median SMI, VATI, SATI, VSR were 40.9cm^2^/m^2^ (IQR, 35.8–46.9), 31.6 cm^2^/m^2^ (IQR, 14.2–48.2), 33.7cm^2^/m^2^ (IQR, 21.8–47.1) and 0.91 (IQR, 0.49–1.39), respectively (Table 2). Sixty-one patients (44.2%) were diagnosed as sarcopenia. The median PFS was 6.5 months (95%CI 5.1–8.2) and the median OS was 15.2 months (95%CI 11.2–19.0).

**Table 1:** Patient Characteristics

	Total cohort (*n* = 138)	SMI change rate ≥ -3.5% (*n* = 84)	SMI change rate < -3.5% (*n* = 54)	*p*-value
Age	67.5 (59.7–74)	69 (61.2–74.7)	66 (59–72.2)	0.12
Age ≥ 75 years old	29 (21.0)	21 (25.0)	8 (14.8)	0.14
Male sex	80 (58.0)	43 (51.2)	37 (68.5)	0.04
ECOG Performance status 0/1/2	76/61/1 (55.1/44.2/0.7)	47/36/1 (56.0/42.9/1.1)	29/25/0 (53.7/46.3/0)	0.57
BMI, kg/m^2^	21.5 (19.5–23.6)	21.3 (19.2–23.6)	21.8 (20.5–23.7)	0.51
Metastasis	97 (70.3)	62 (73.8)	35 (64.8)	0.26
Liver	62 (44.9)	41 (48.8)	21 (38.9)	0.25
Lung	20 (14.5)	14 (16.7)	6 (11.1)	0.35
Lymph node	38 (27.5)	28 (33.3)	10 (18.5)	0.05
Peritoneal dissemination	21 (15.2)	10 (11.9)	11 (20.4)	0.18
Biliary drainage before chemotherapy	31 (22.5)	11 (13.1)	20 (37.0)	< 0.01
CA19-9, U/ml	678.5 (105.2–4098)	531 (55.7–3868.7)	1064.5 (200.2–4546.2)	0.33
NLR	2.9 (2.1–4.1)	2.8 (2–3.9)	3 (2.4–4.7)	0.31
PLR	176.9 (128.9–246.6)	164.2 (128.3–229.0)	192.8 (134.9–256.5)	0.40
mGPS, 0	61 (44.9)	36 (43.9)	25 (46.3)	0.78
CCI, ≥ 3	15 (10.9)	8 (9.5)	7 (13.0)	0.52
SMI, cm^2^/m^2^	40.9 (35.8–46.9)	39.8 (35.2–45.2)	43.8 (36.3–50.0)	0.02
VATI, cm^2^/m^2^	31.6 (14.2–48.2)	31.6 (13.4–46.1)	31.3 (14.6–55.4)	0.08
SATI, cm^2^/m^2^	33.7 (21.8–47.1)	33.5 (21.1–45.2)	34.3 (24.9–48.6)	0.93
VSR	0.91 (0.49–1.39)	0.81 (0.48–1.36)	0.97 (0.51–1.45)	0.40
Sarcopenia*	61 (44.2)	40 (47.6)	21 (38.9)	0.31
Interval between pretreatment and initial evaluation CT	61.5 (24–109)	62 (55–71)	60 (52–69.2)	0.11

Numbers are shown in n (%) or median (interquartile range [IQR]). *Defined as male SMI < 42 cm^2^/m^2^ and female SMI < 38 cm^2^/m^2^ based on the criteria proposed by the Hepatology Society of Japan

*BMI* Body mass index, *CCI* Charlson comorbidity index, *ECOG* Eastern Cooperative Oncology Group, *mGPS* modified Glasgow Prognostic Score, *NLR* Neutrophil/lymphocyte ratio, *PLR* Platelet/lymphocyte ratio, *SATI* subcutaneous adipose tissue index, *SMI* skeletal muscle mass index, *VATI* visceral adipose tissue index, *VSR* visceral-to-subcutaneous fat area ratio

**Table 2:** Body composition according to the SMI change rate

	Total (*n* = 138)	SMI change rate ≥ -3.5% (*n* = 84)	SMI change rate < -3.5% (*n* = 54)	*p*-value
SMI change rate, %	-2.1 (-6.5–2.1)	0.55 (-1.7–4.9)	-8.3 (-12.5- -5.6)	< 0.01
SMI before chemotherapy, cm^2^/m^2^	40.9 (35.8–46.9)	39.8 (35.2–45.2)	43.8 (36.3–50.0)	0.02
SMI at initial evaluation, cm^2^/m^2^	40.4 (35.2–45.2)	41.0 (36.1–45.3)	39 (32.5–44.1)	0.11
VATI before chemotherapy, cm^2^/m^2^	31.6 (14.2–48.2)	31.6 (13.4–46.1)	31.3 (14.6–55.4)	0.08
VATI at initial evaluation, cm^2^/m^2^	23.1 (11.2–43.2)	26.6 (10.7–43.8)	21.9 (11.2–43.2)	0.76
SATI before chemotherapy, cm^2^/m^2^	33.7 (21.8–47.1)	33.5 (21.1–45.2)	34.3 (24.9–48.6)	0.93
SATI at initial evaluation, cm^2^/m^2^	28.7 (17.3–41.3)	28.3 (18.8–43.1)	29.3 (15.2–40.8)	0.11
VSR before chemotherapy	0.91 (0.49–1.39)	0.81 (0.48–1.36)	0.97 (0.51–1.45)	0.40
VSR at initial evaluation	0.85 (0.56–1.30)	0.83 (0.53–1.27)	0.87 (0.56–1.35)	0.81
Sarcopenia* before chemotherapy	61 (44.2)	40 (47.6)	21 (38.9)	0.31
Sarcopenia* at initial evaluation	66 (47.8)	34 (40.5)	32 (59.3)	0.03

Numbers are shown in n (%) or median (interquartile range [IQR]). *Defined as male SMI < 42 cm^2^/m^2^ and female SMI < 38 cm^2^/m^2^ based on the criteria proposed by the Hepatology Society of Japan

*BMI* Body mass index, *CCI* Charlson comorbidity index, *ECOG* Eastern Cooperative Oncology Group, *mGPS* modified Glasgow Prognostic Score, *SATI* subcutaneous adipose tissue index, *SMI* Skeletal muscle mass index, *VATI* visceral adipose tissue index, *VSR* Visceral-to-subcutaneous fat area ratio

### SMI change rate and clinical outcomes

**Fig. 3:**
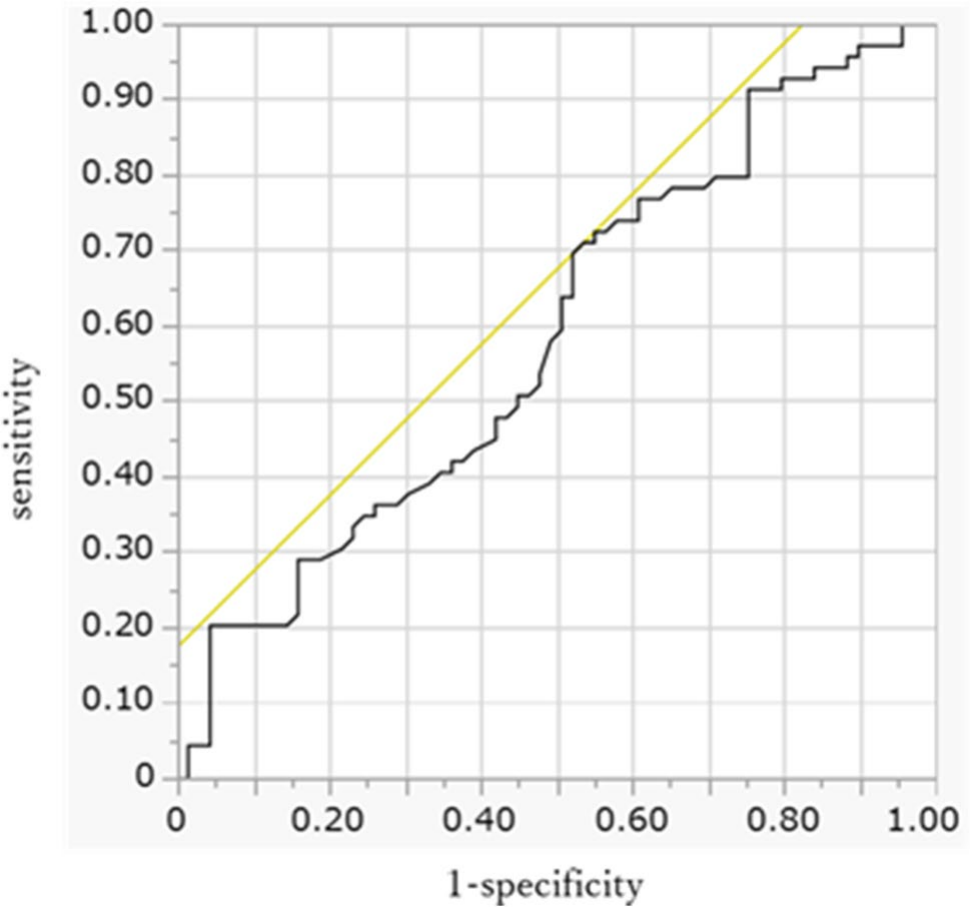
Receiver operating characteristic curve with SMI change rate

**Fig. 4:**
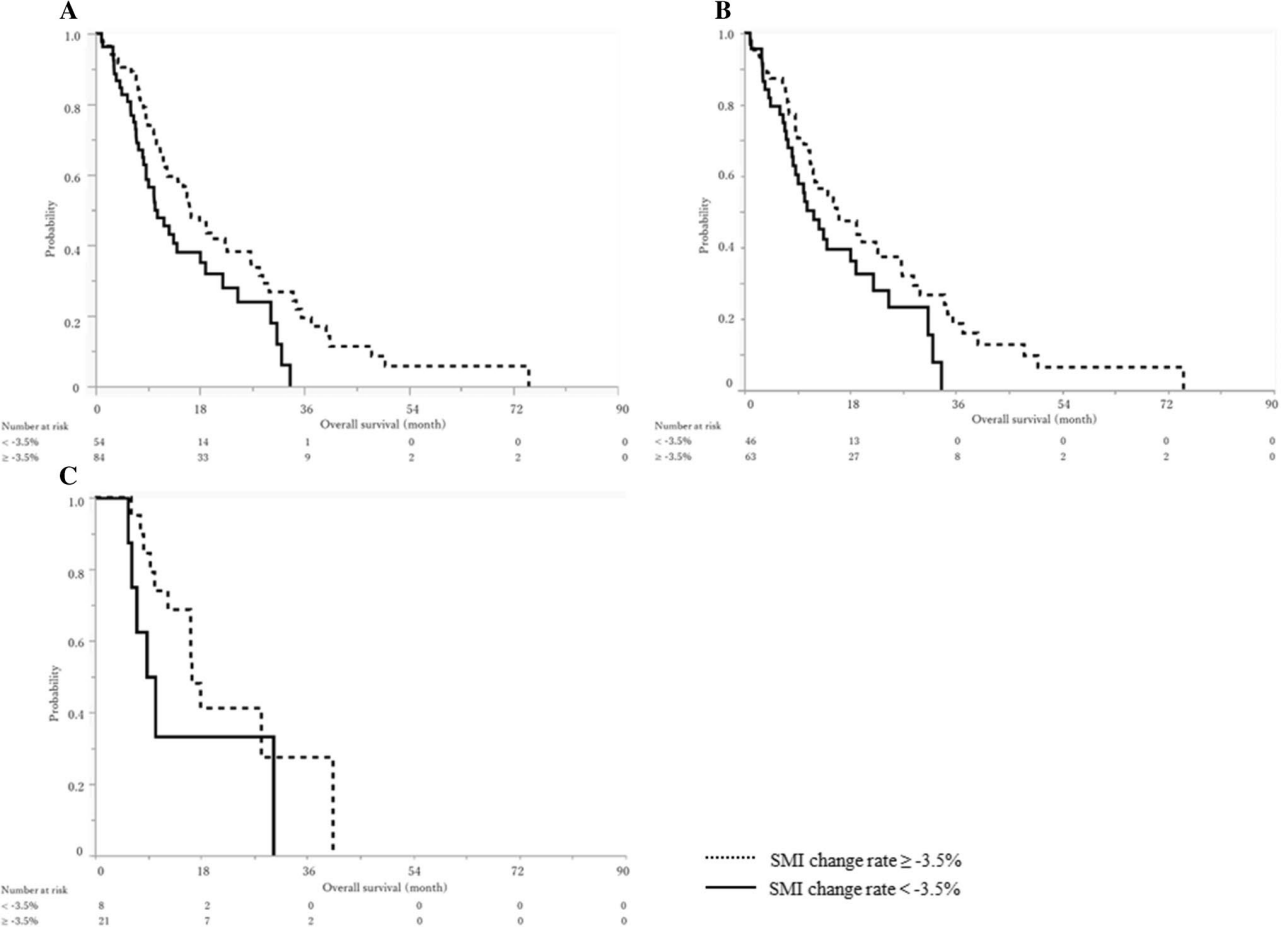
Overall survival according to the SMI change rate. Solid lines indicate SMI change rate < -3.5% and broken lines indicate SMI change rate ≥ -3.5%. **A**. Overall survival of the total cohort. The median overall survival was 10.3 months (95%CI, 8.3–18.1) for SMI change rate < -3.5% and 16.3 months (95%CI, 12.3–22.7) for SMI change rate ≥ -3.5% (*P* = 0.01). **B**. Overall survival in non-elderly (< 75 years old) patients. The median overall survival was 11.8 months (95%CI, 8.2–19.0) for SMI change rate < -3.5% and 15.8 months (95%CI, 11.2–22.7) for SMI change rate ≥ -3.5% (*P* = 0.07). **C**. Overall survival in elderly (≥ 75 years old) patients. The median overall survival was 9.5 months (95%CI, 5.5–30.2) for SMI change rate < -3.5% and 16.5 months (95%CI, 10.2–40.4) for SMI change rate ≥ -3.5% (*P* = 0.11)

By creating ROC curve with a dichotomous variable divided by the median OS as the dependent variable, the cutoff value for SMI change rate was set at -3.5% (Fig. 3). The median OS in the total cohort was 16.3 months (95%CI 12.3–22.7) and 10.3 months (95%CI 8.3–18.1) in SMI change rate ≥ -3.5% and < -3.5% groups (*P* = 0.01, Fig. 4A).

Patient characteristics divided by SMI change rate are shown in Table 1. The rates of male sex and biliary drainage were significantly higher in SMI change rate < -3.5% group. Body composition before chemotherapy and at the initial evaluation is shown in Table 2. The median SMI before chemotherapy was higher in SMI change rate < -3.5% group: 39.8 and 43.8 cm^2^/m^2^ (*P* = 0.02), but the difference was not significant at the initial evaluation. The rate of sarcopenia at the initial evaluation was significantly higher in SMI change rate < -3.5% group: 40.5% and 59.3% (*P* = 0.03).

**Fig. 5:**
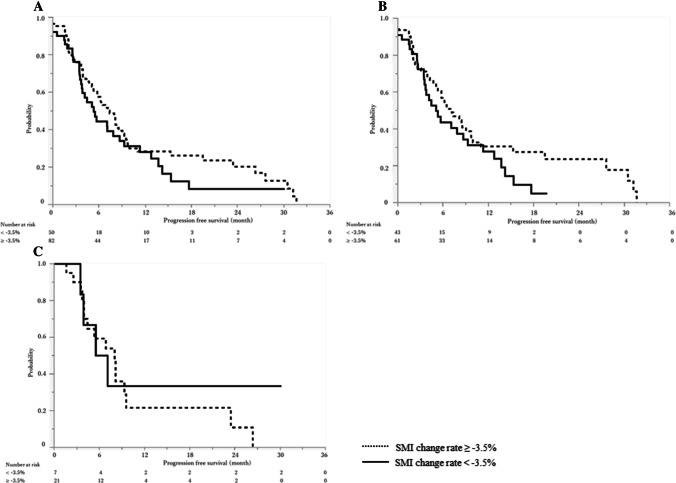
Progression free survival according to the SMI change rate. Solid lines indicate SMI change rate < -3.5% and broken lines indicate SMI change rate ≥ -3.5%. **A**. Progression free survival of the total cohort. The median PFS was 5.4 months (95%CI, 3.7–8.7) for SMI change rate < -3.5% and 7.2 months (95%CI, 5.3–9.1) for SMI change rate ≥ -3.5% (p = 0.24). **B**. Progression free survival in non-elderly (< 75 years old) patients. The median progression free survival was 5.1 months (95%CI, 3.5–8.7) for SMI change rate < -3.5% and 7.2 months (95%CI, 5.1–9.8) for SMI change rate ≥ -3.5% (*P* = 0.16). **C**. Progression free survival in elderly (≥ 75 years old) patients. The median progression free survival was 6.3 months (95%CI, 3.5-NA) for SMI change rate < -3.5% and 8.0 months (95%CI, 4.0–9.6) for SMI change rate ≥ -3.5% (*P* = 0.66). CI; confidence interval, SMI; Skeletal muscle mass index

There were no significant differences in objective response (*P* = 0.55): RR was 23.8% and 16.7% and DCR was 89.3% and 81.5% in SMI change rate ≥ -3.5% and < -3.5% groups (Table 3). The median PFS by SMI change rate in the total cohort was not significantly different: 7.2 months (95%CI 5.3–9.1) and 5.4 months (95%CI 3.7–8.7) in SMI change rate ≥ -3.5% and < -3.5% groups, respectively (*P* = 0.24, Fig. 5A).

**Table 3:** Treatment outcomes according to the SMI change rate

	Total (*n* = 138)	SMI change rate ≥ -3.5% (*n* = 84)	SMI change rate < -3.5% (*n* = 54)	*p*-value
Number of cycles	7.5 (4–12)	8 (5–12.7)	6 (2.7–10.2)	0.01
RDI for the first two cycles, %	69.2 (56.7–83.3)	66.7 (57.5–83.3)	70 (56.7–83.3)	0.45
Dose reduction at 1st cycle	90 (65.2)	54 (64.3)	36 (66.7)	0.77
ETS, %	12 (0–22.7)	13.6 (1.8–23.1)	9.2 (0–21.4)	0.44
Best response				0.55
Complete response	1 (0.7)	1 (1.2)	0	
Partial response	28 (20.3)	19 (22.6)	9 (16.7)	
Stable disease	90 (65.2)	55 (65.5)	35 (64.8)	
Progression disease	13 (9.4)	6 (7.1)	7 (13.0)	
Not evaluable	6 (4.4)	3 (3.6)	3 (5.5)	
Response rate, %	21.0	23.8	16.7	0.30
Disease control rate, %	86.2	89.3	81.5	0.19
Reasons for discontinuation				
Disease progression	95 (68.9)	60 (71.4)	35 (64.8)	0.41
Serious adverse event	17 (12.3)	11 (13.1)	6 (11.1)	0.72
Poor general condition	13 (9.4)	6 (7.1)	7 (13.0)	0.25
Discontinue at initial evaluation	21 (15.2)	8 (9.5)	13 (24.1)	0.02
Introduction of 2nd line treatment	105 (79.0)	66 (80.5)	39 (76.5)	0.58

Numbers are shown in n (%) or median (interquartile range [IQR]). *ETS* Early tumor shrinkage, *RDI* relative dose intensity

In terms of safety, the incidences of AEs were comparable between two groups, other than all grades neutropenia (Table 4). However, SMI change rate < -3.5% group had experienced more discontinuations at initial evaluation (*P* = 0.02), and fewer total cycles of chemotherapy (*P* = 0.01) compared to SMI change rate ≥ -3.5% group.

**Table 4:** Adverse effects according to the SMI change rate

	All grades			Grade ≥ 3		
	SMI change rate ≥ -3.5%	SMI change rate < -3.5%	*p*-value	SMI change rate ≥ -3.5%	SMI change rate < -3.5%	*p*-value
Hematologic						
Neutropenia	75 (89.3)	39 (72.2)	0.01	53 (63.1)	30 (55.6)	0.37
Thrombocytopenia	49 (58.3)	25 (46.3)	0.20	4 (4.8)	0	0.07
Anemia	72 (85.7)	45 (83.3)	0.52	12 (14.3)	3 (5.6)	0.14
Non-hematologic						
Vomiting	5 (6.0)	1 (1.9)	0.28	0	0	
Nausea	18 (21.4)	5 (9.3)	0.08	2 (2.4)	0	0.22
Anorexia	32 (38.1)	21 (38.9)	0.60	2 (2.4)	1 (1.9)	0.59
Fatigue	18 (21.4)	13 (24.1)	0.57	3 (3.6)	0	0.13
Diarrhea	12 (14.3)	7 (13.0)	0.58	1 (1.2)	1 (1.9)	0.57
Constipation	35 (41.7)	19 (35.2)	0.43	0	0	
Peripheral neuropathy	46 (54.8)	21 (38.9)	0.10	3 (3.6)	3 (5.6)	0.52

Numbers are shown in *n* (%)

### Prognostic factors for PFS and OS

The results of univariable and multivariable analyses of PFS and OS are shown in Tables 5A, B. In the multivariable analysis, CA19-9 (HR 2.12, 95% CI 1.34–3.36, *P* < 0.01) and mGPS (HR 1.58, 95% CI 1.02–2.44, *P* = 0.03) were significant prognostic factors for PFS. Meanwhile, CA19-9 (HR 3.34, 95% CI 2.00–5.57, *P* < 0.01), PLR (HR 1.68, 95%CI 1.01–2.78, *P* = 0.04), mGPS (HR 2.32, 95%CI 1.47–3.65, *P* < 0.01) and RDI up to 2 cycles (HR 2.21, 95%CI 1.42–3.46, *P* < 0.01) were significantly prognostic factors for OS. SMI change rate (HR 1.47, 95%CI 0.95–2.28, *P* = 0.08) and ETS (HR 1.53, 95%CI 0.94–2.49, *P* = 0.08) was also associated with OS, though statistically not significant. Neither sarcopenia before chemotherapy nor sarcopenia at initial evaluation was significantly associated with PFS or OS.

**Table 5:** Prognostic factors for progression free survival and overall survival

	Univariable analysis		Multivariable analysis	
	HR (95% CI)	*p*-value	HR (95% CI)	*p*-value
5A. Progression free survival				
Age ≥ 75y	0.93 (0.56–1.54)	0.79		
Male Sex	0.86 (0.57–1.30)	0.49		
ECOG Performance status 1, 2	1.00 (0.66–1.51)	0.98		
BMI < 22	1.10 (0.73–1.65)	0.64		
Metastatic disease	1.52 (0.96–2.40)	0.07	1.20 (0.74–1.96)	0.45
CA19-9 ≥ 500 U/ml	2.37 (1.54–3.65)	< 0.01	2.12 (1.34–3.36)	< 0.01
NLR ≥ 3.2	1.41 (0.93–2.13)	0.09	1.20 (0.75–1.92)	0.43
PLR ≥ 195	1.61 (1.05–2.48)	0.02	1.25 (0.77–2.04)	0.36
mGPS 1, 2	1.72 (1.12–2.63)	0.01	1.58 (1.02–2.44)	0.03
CCI ≥ 3	1.52 (0.77–2.98)	0.21		
Biliary drainage before chemotherapy, Yes	1.01 (0.61–1.67)	0.94		
Sarcopenia* before chemotherapy, Yes	1.12 (0.74–1.69)	0.59		
VSR before chemotherapy, Male ≥ 1.26, Female ≥ 0.52	0.86 (0.57–1.30)	0.48		
Sarcopenia at initial evaluation, Yes	1.08 (0.72–1.63)	0.68		
VSR at initial evaluation, Male ≥ 1.29, Female ≥ 0.56	1.23 (0.81–1.85)	0.32		
SMI change rate < -3.5%	1.28 (0.84–1.96)	0.24		
RDI < 69.2%	1.20 (0.79–1.81)	0.37		
Dose reduction at 1st cycle, Yes	0.85 (0.55–1.30)	0.45		
ETS < 20%	1.22 (0.78–1.92)	0.36		
5B. Overall survival				
Age ≥ 75y	0.92 (0.55–1.55)	0.77		
Male Sex	1.06 (0.71–1.60)	0.74		
ECOG Performance status 1, 2	1.02 (0.68–1.54)	0.89		
BMI < 22	1.05 (0.70–1.57)	0.79		
Metastatic disease	1.69 (1.07–2.68)	0.02	1.24 (0.76–2.02)	0.36
CA19-9 ≥ 500 U/ml	3.62 (2.22–5.88)	< 0.01	3.34 (2.00–5.57)	< 0.01
NLR ≥ 3.2	1.58 (1.05–2.37)	0.02	1.25 (0.76–2.05)	0.36
PLR ≥ 195	2.22 (1.44–3.41)	< 0.01	1.68 (1.01–2.78)	0.04
mGPS 1, 2	1.98 (1.30–3.02)	< 0.01	2.32 (1.47–3.65)	< 0.01
CCI ≥ 3	1.14 (0.57–2.30)	0.69		
Biliary drainage before chemotherapy, Yes	1.27 (0.78–2.04)	0.32		
Sarcopenia* before chemotherapy, Yes	1.13 (0.75–1.69)	0.54		
VSR before chemotherapy, Male ≥ 1.26, Female ≥ 0.52	0.81 (0.54–1.22)	0.33		
Sarcopenia at initial evaluation, Yes	1.29 (0.85–1.94)	0.22		
VSR at initial evaluation, Male ≥ 1.29, Female ≥ 0.56	0.95 (0.64–1.42)	0.83		
SMI change rate < -3.5%	1.64 (1.08–2.52)	0.02	1.47 (0.95–2.28)	0.08
RDI < 69.2%	1.52 (1.00–2.31)	0.04	2.21 (1.42–3.46)	< 0.01
Dose reduction at 1st cycle, Yes	1.19 (0.77–1.84)	0.41		
ETS < 20%	1.42 (0.90–2.23)	0.12	1.53 (0.94–2.49)	0.08

*BMI* Body mass index, *CCI* Charlson comorbidity index, *CI* confidence interval, *ECOG* Eastern Cooperative Oncology Group, *ETS* early tumor shrinkage, *mGPS* modified Glasgow Prognostic Score, *HR* hazard ratio, *NLR* Neutrophil/lymphocyte ratio, *PLR* Platelet/lymphocyte ratio, *RDI* Relative dose intensity, *SMI* skeletal muscle mass index, *VSR* visceral-to-subcutaneous fat area ratio

^*^Defined as male SMI < 42 cm^2^/m^2^ and female SMI < 38 cm^2^/m^2^ based on the criteria proposed by the Hepatology Society of Japan

### Exploratory analyses of body composition by age

Twenty-nine patients (21.0%) were ≥ 75 years old in our cohort. There were no significant differences in RR (19.3% vs. 27.6%, *P* = 0.34), the median PFS (6.3 vs. 7.1 months, *P* = 0.79) and the median OS (14.1 vs. 16.3 months, *P* = 0.77) between non-elderly (< 75 years old) and elderly (≥ 75 years old) patients. When body composition was compared between non-elderly and elderly patients (Table 6), VATI both before chemotherapy and at initial evaluation was significantly higher in elderly patients. There were no significant differences in sarcopenia (44.0% and 44.8%, *P* = 0.93) or SMI change rates (-2.4% and -1.8%, *P* = 0.23) in non-elderly and elderly patients. The median PFS was 5.1 and 7.2 months in SMI change rate < -3.5% and SMI change rate ≥ -3.5% groups in non-elderly patients (*P* = 0.16, Fig. 5B), while it was 6.3 and 8.0 months in SMI change rate < -3.5% and SMI change rate ≥ -3.5% groups in elderly patients (*P* = 0.66, Fig. 5C). SMI change rate was associated with OS, though not statistically significant. While the median OS was 11.8 and 15.8 months in SMI change rate < -3.5% and SMI change rate ≥ -3.5% groups in non-elderly patients (*P* = 0.07, Fig. 4B), it was 9.5 and 16.5 months for SMI change rate < -3.5% and SMI change rate ≥ -3.5% groups in elderly patients (*P* = 0.11, Fig. 4C).

**Table 6:** Body composition according to the age

	< 75 years old (*n* = 109)	≥ 75 years old (*n* = 29)	*p*-value
SMI change rate, %	-2.4 (-6.6–1.9)	-1.8 (-6.4–7.1)	0.23
SMI before chemotherapy, cm^2^/m^2^	40.8 (35.5–47.5)	42.3 (35.9–45.8)	0.44
SMI at initial evaluation, cm^2^/m^2^	40.0 (25.1–45.2)	42.0 (35.2–45.1)	0.85
VATI before chemotherapy, cm^2^/m^2^	29.4 (12.1–43.6)	45.0 (27.9–57.8)	< 0.01
VATI at initial evaluation, cm^2^/m^2^	21.5 (10.2–41.3)	35.2 (18–52.5)	0.03
SATI before chemotherapy, cm^2^/m^2^	30.9 (19.6–49.3)	37.4 (29.4–44.5)	0.66
SATI at initial evaluation, cm^2^/m^2^	26.5 (15.5–40.7)	34.0 (26.9–43.4)	0.49
VSR before chemotherapy	0.84 (0.48–1.32)	1.13 (0.76–1.61)	0.16
VSR at initial evaluation	0.81 (0.48–1.23)	0.93 (0.71–1.44)	0.27
Sarcopenia* before chemotherapy	48 (44.0)	13 (44.8)	0.93
Sarcopenia* at initial evaluation	54 (49.5)	12 (41.4)	0.43

Numbers are shown in n (%) or median (interquartile range [IQR]). *Defined as male SMI < 42 cm^2^/m^2^ and female SMI < 38 cm^2^/m^2^ based on the criteria proposed by the Hepatology Society of Japan

*BMI* Body mass index, *CCI* Charlson comorbidity index, *ECOG* Eastern Cooperative Oncology Group, *mGPS* modified Glasgow Prognostic Score, *SATI* subcutaneous adipose tissue index, *SMI* Skeletal muscle mass index, *VATI* visceral adipose tissue index, *VSR* Visceral-to-subcutaneous fat area ratio

## Discussion

In this retrospective study, we found that early skeletal muscle mass decline was associated with shorter OS in patients receiving first-line GnP for unresectable PC. On the other hands, sarcopenia before chemotherapy was not associated with OS. Our study results suggested early decline of SMI after introduction of chemotherapy rather than the value of SMI before chemotherapy might be prognostic of survival in patients with unresectable PC.

Sarcopenia as one of prognostic factors in patients with cancer is increasingly reported in various cancers. Recent studies suggested the role of sarcopenia in patients receiving palliative chemotherapy for PC. In our cohort, sarcopenia was observed in 44.2% at the time of diagnosis, which was similar to that of previous reports [7, 16]. While some studies suggested association of sarcopenia at diagnosis with prognosis [12, 14, 25], others reported change in body composition was associated with survival [10–12, 26]. Sarcopenia before chemotherapy was not associated with PFS or OS in our cohort, by using the criteria developed by the Hepatology Society of Japan based on the AWGS criteria (male SMI < 42 cm^2^/m^2^ and female SMI < 38 cm^2^/m^2^) [23]. However, SMI change up to initial evaluation was associated with OS, suggesting body composition change can be predictive of prognosis in patients receiving palliative chemotherapy for PC. Interestingly, our definition of SMI decline > -3.5% was not associated with tumor response or PFS. The reason for discrepancy between PFS and OS is unclear but the similar outcomes were also observed in elderly patients receiving GnP chemotherapy [16].

In terms of safety, it was suggested that SMI change was not significantly associated with either AEs, other than all grades neutropenia, or RDI up to first 2 cycles of chemotherapy. Since reduced RDI was associated with poor survival, the maintenance of RDI is as important as the control of severe AEs, as previous studies reported the association of RDI with efficacy of FOLFIRINOX for PC [27, 28]. In our study, though 2-cycle RDI was comparable, discontinuation of chemotherapy after initial evaluation (24.1% and 9.5%) and discontinuation due to poor condition (13.0% and 7.1%) were more often encountered in SMI change rate ≥ -3.5% group compared to SMI change rate < -3.5% group. As a result, the number of cycles was higher in SMI change rate ≥ -3.5% group. Thus, sarcopenia during chemotherapy can lead to cessation of chemotherapy due to the deteriorated patient condition and non-chemotherapeutic support to prevent sarcopenia might improve clinical outcomes of palliative chemotherapy in PC.

Nutritional support has been increasingly investigated in the field of oncology. Anamorelin, an oral ghrelin-like agent, reportedly improved body weight and anorexia-related symptoms in cancer patients [29] and we also reported that insufficient protein intake was a poor prognostic factor in patients with unresectable PC receiving chemotherapy [30]. Nutritional interventions such as nutritional supplements [31] or pancreatic exocrine replacement treatment [32, 33] might also affect body composition. Thus, we should further investigate whether those nutritional interventions would improve sarcopenia during chemotherapy and lead to the improved prognosis or not.

Age itself can affect body composition and its impact on chemotherapy might differ by age. However, in our exploratory analyses, the associations of SMI change were comparable between elderly and non-elderly patients. The median OS tended to be longer in cases with SMI decline ≥ -3.5%, regardless of age. Meanwhile, a previous study of pancreatic cancer receiving GnP chemotherapy reported that sarcopenia at diagnosis was associated with poor OS only in elderly (> 70 years old) patients [16]. We previously reported comorbidity, rather than age, was an important prognostic factors in gemcitabine-based chemotherapy [34]. Recently, the importance of cognitive assessment is also reported in elderly patients [35, 36]. The relation of age, comorbidity and body composition can be multifactorial and more comprehensive evaluation in a large prospective cohort is warranted.

Our study had several limitations. Firstly, this was a retrospective study at a single academic center and the selection bias was inevitable. For example, the rate of sarcopenia at diagnosis of PC was similar between elderly and non-elderly patients. Elderly patients who could receive GnP might be a selected population in a good clinical condition. Thus, our study results need to be validated in the external cohort. Secondly, definition of sarcopenia using CT scan have not been established. The AWGS 2019 definition uses grip strength, physical function (walking speed, 5 times stand up, short physical performance battery) and skeletal muscle mass measurement by dual energy X-ray absorptiometry or bioelectrical impedance analysis to determine sarcopenia [37]. We applied the criteria for sarcopenia by the Hepatology Society of Japan since this was retrospective study. Definition of sarcopenia in cases with malignancy including PC receiving palliative chemotherapy needs further investigation.

In conclusion, short-term decline of skeletal muscle mass was associated with poor OS in patients receiving GnP for unresectable PC. Further investigation is warranted whether the maintenance of skeletal muscle mass by nutritional support or medications would improve prognosis or not.

## Data Availability

The datasets generated during and/or analysed during the current study are not publicly available but are available from the corresponding author on reasonable request.

## Abbreviations

AEs: Adverse effects
BMI: Body mass index
CA19-9: Carbohydrate antigen 19–9
CCI: Charlson comorbidity index
CI: Confidence interval
DCR: Disease control rate
ECOG PS: Eastern Cooperative Oncology Group performance status
ETS: Early tumor shrinkage
GnP: Gemcitabine and nab-paclitaxel
HR: Hazard ratio
HU: Hounsfield unit
IQR: Interquartile range
mGPS: Modified Glasgow Prognostic Score
NLR: Neutrophil/lymphocyte ratio
OS: Overall survival
PC: Pancreatic cancer
PFS: Progression free survival
PLR: Platelet/lymphocyte ratio
RDI: Relative dose intensity
ROC: Receiver operating characteristic
RR: Response rate
SATI: Subcutaneous adipose tissue index
SMI: Skeletal muscle mass index
VATI: Visceral adipose tissue index
VSR: Visceral-to-subcutaneous fat area ratio
